# Five Steps to Inject Transformative Change into the Post-2020 Global Biodiversity Framework

**DOI:** 10.1093/biosci/biab013

**Published:** 2021-03-03

**Authors:** R Edward Grumbine, Jianchu Xu

**Affiliations:** Chinese Academy of Sciences President's International Fellowship Initiative, Centre for Mountain Futures, Kunming Institute of Botany, Kumming, China; East and Central Asia Office, World Agroforestry Centre, Kunming, China, and is the director of the Centre for Mountain Futures and a professor at the Kunming Institute of Botany, Chinese Academy of Sciences, in Kunming, China

**Keywords:** biodiversity conservation, Convention on Biological Diversity, transformative change, Post-2020 Global Biodiversity Framework

## Abstract

Accelerating declines in biodiversity and unmet targets in the Convention on Biological Diversity's 2010–2020 Strategic Plan for Biodiversity are stimulating widespread calls for transformative change. Such change includes societal transitions toward sustainability, as well as in specific content of the CBD's draft Post-2020 Global Biodiversity Framework. We summarize research on transformative change and its links to biodiversity conservation, and discuss how it may influence the work of the CBD. We identify five steps to inject transformative change into the design and implementation of a new post-2020 framework: Pay attention to lessons learned from transitions research, plan for climate change, reframe area-based conservation, scale up biodiversity mainstreaming, and increase resources. These actions will transform the very nature of work under the CBD; a convention based on voluntary implementation by countries and facilitated by international administrators and experts must now accommodate a broader range of participants including businesses, Indigenous peoples, and multiple nonstate actors.

Attempts to reverse accelerating, broad-based declines in biodiversity are at a crossroads in 2021. Comprehensive global assessment shows that 14 of 18 important trends in biodiversity are negative (Díaz et al. 2019). The majority of Aichi targets in the Convention on Biological Diversity's (CBD) 2010–2020 Strategic Plan for Biodiversity have not been met, extending unsuccessful efforts of the previous decade (CBD 2020a). And there has been limited progress to date on achieving biodiversity-related targets in the United Nations (UN) Sustainable Development Goals (SDGs) (UN 2019).

The CBD grew out of global negotiations that culminated in 1992 at the UN Conference on Environment and Development. The convention became international law in 1993 with the authority to prescribe legally binding protocols on signatory parties to conserve biodiversity, sustain its use, and support fair and equitable benefit sharing of genetic resources (Harrop and Pritchard 2011). However, political compromises during its construction and implementation have resulted in a soft power administrative body, the CBD secretariat, and member states that have historically favored aspirational policy guidance over legal obligations (Raustiala 1997).

Now, with 20 years of work leading to such minimal progress in biodiversity protection, many have called for transformative change in the content of the CBD's draft 2020–2030 Global Biodiversity Framework (hereafter, the Post-2020 Framework). This framework is under negotiation in advance of its finalization at the upcoming conference of the parties (COP) 15 in Kunming, China (currently rescheduled for later in 2021 because of the SARS-CoV-2 virus). Transformative change has been defined as “fundamental, system-wide reorganization across technological, economic, and social factors, making sustainability the norm rather than the altruistic exception” (Díaz et al. 2019, p. 7). The current draft of the Post-2020 Framework supports this view declaring that urgent action “is required to transform economic, social and financial models so that… trends that have exacerbated biodiversity loss will stabilize [by 2030], and allow for the recovery of natural ecosystems” by 2050 (CBD 2020b).

But promoting such transformation is a tremendous challenge to fit into efforts for biodiversity conservation to 2030 and beyond. A glance at several of the targets in the draft reveals the depth of change under discussion. These include expanding area-based conservation to at least 30% of Earth's terrestrial and marine ecosystems from today's respective 17% and 10%, eliminating hundreds of billions of dollars in subsidies from nation-state budgets that negatively affect biodiversity, applying biodiversity values to every scale of environmental assessment from local to international levels, and encouraging people across the planet to become sustainable consumers (CBD 2020b). It is clear that transitions as represented by these goals would require that conservation scientists and practitioners expand their traditional concerns with protected areas, threatened species, and management of natural resources to whole-societal transformative change (Ellis and Mehrabi 2019). We agree with many scientists and advocates that this must be done, despite the profound challenges that are embedded in the meaning of transformative change and its practical application to conservation. The question is *how*?

In the present article, we highlight five steps to inject transformative change into the work of the CBD and implementation of the Post-2020 Framework. Our intent is to stimulate movement away from abstract calls for change toward more strategic implementation of on-the-ground actions using ideas from transformative change research as it relates to protecting biodiversity for people and nature. We present these five steps after careful reading of 239 publications selected after review of 781 papers on biodiversity conservation and transformative change/sustainable transitions as this literature addresses conservation into the post-2020 period. Using the Google Scholar and Web of Science databases, we performed multiple searches beginning with “CBD” and the “Post-2020 Framework” followed by key descriptors from the 20 targets in the Post-2020 Framework (CBD 2020b). We also surveyed 47 publically available documents produced by the CBD from 2010 to January 2021 that discuss general CBD goals and crafting the Post-2020 Framework. From this search, we identified three targets that were repeatedly referred to in this literature as key to achieving biodiversity goals. We added in the metatargets of transformative change and planning for climate impacts because these two steps deeply influence all CBD work.

## Five steps to inject transformative change into biodiversity conservation

Numerous steps can be taken to improve success in biodiversity conservation, and, given the breadth represented by the Post-2020 Framework targets, it is apparent that other scholars might have selected different steps to focus on; we do not suggest that other post-2020 targets are unimportant. But it is clear that without greater attention to how biodiversity work can benefit from what has already been learned about how societal transitions occur, addressing climate change across all post-2020 targets, transformative reframing of traditional conservation to embrace both protected areas and surrounding unprotected lands (target 2), heightened efforts to incorporate biodiversity into the very structure of environmental planning (target 13), and a substantial scaling up of financial resources to accomplish the above (target 18), next-generation conservation will be considerably diminished. Simply stated, fulfilling the goals of the CBD will not occur without strategic learning about societal change, explicit incorporation of climate concerns into conservation; future-forward reframing of what *protected* means, mainstreaming environmental values into multiple rules and regulations, and finding the money to pay for it all.

Although the five steps we highlight are critical to post-2020 outcomes, none of them are new proposals; most of them have been and continue to be the focus of much work within the CBD and the conservation community. For years, however, progress toward making these steps manifest has been minimal even as declines in biodiversity have accelerated (Díaz et al. 2019). We acknowledge that the CBD secretariat is a UN body facilitating 196 signatory nations using voluntary compliance absent any consensual global governance mechanism. In grappling with transformative change, the CBD, like any international body, is subject to competing interests, unequal power relationships, and institutional inertia (Wyborn et al. 2020). We also understand that transformative change for people and nature requires much more than we have been able to achieve thus far.

## Step 1: Pay attention to lessons learned about transformative change

Calling for transformative change is easy to do given accelerating biodiversity loss, but what do we already know about societal transformations after more than two decades of research? Below, we summarize key points that appear in the literature (for general overviews, see O'Brien 2012, Loorbach et al. 2017, and Scoones et al. 2020). Transformative change can be defined in several ways (see Díaz et al. 2019 and CBD 2020b above) but, in general, involves transition from one system to another through dynamic, nonlinear, often disruptive processes (Patterson et al. 2017). Change often involves sparking innovation and then diffusing new ways of thinking and acting into the larger system until older structures fade away, leading to whole-of-society transformation. Few, if any, of these processes are linear; all of them are deeply political (Blythe et al. 2018). Transitions research aims to understand how various actors block or support transformations, so that desired change (e.g., toward greater support and better outcomes for biodiversity) may be anticipated, advanced, and, possibly, accelerated.

Unlike researchers working on energy and food systems transitions (see Park et al. 2012, Victor et al. 2019, Dorninger et al. 2020), conservation workers have done comparatively little to apply lessons learned about transformative change to biodiversity strategies as was indicated by the few citations to this work that we found in peer-reviewed papers. Stimulated by increasing climate impacts, more conservation researchers are arguing for new adaptive management strategies as species and ecosystems change (see step 2 below). But this work is only beginning to reference specific lessons learned from the societal transitions literature. The CBD secretariat also does not appear to have engaged with the transformative change literature, nor has it encouraged much experimentation with practical applications highlighted in this research. To enhance implementation of the Post-2020 Framework, this must change.

How do you support activities that allow for innovation to emerge out of the status quo? Three broad lines of transitions research—structural, systemic, and enabling—suggest that diverse kinds of knowledge must be recognized, multiple solutions to problems should be experimented with, and that politics and power relationships must be explicitly engaged with (Turnheim et al. 2015, Scoones et al. 2020). Common strategies that encourage transitions include reframing problems to stimulate innovation (Raymond et al. 2013), supporting transdisciplinary knowledge coproduction (Clark et al. 2016, Norström et al. 2020), identifying institutions that are willing to support new projects (Heikkila and Gerlak 2019), strategically prioritizing actions because resources will likely be insufficient to accomplish all tasks (Kern and Rogge 2018), and employing multiple pilot projects led by coalitions of diverse participants to learn what works and what does not before scaling up actions (table 1; Tengo et al. 2017). Identifying leverage points which may reveal institutions, actors, and ideas that serve as barriers to transformations is important in the design of pathways to effect sustainable transitions (Meadows 1999, Sharpe and Lenton 2021). Social justice and equity also must be accounted for in transformative change (Bennett et al. 2020).

**Table 1.** Transformative change strategies for biodiversity conservation.

Strategy	Applications	References
Reframe problems	Redefine working networks for area-based conservation	Dudley et al. 2018, Mitchell et al. 2021
	Include private sector funding into conservation	WEF 2020
Support knowledge coproduction	Create diverse working groups for marine protected areas	Tittensor et al. 2019
Identify partners	Formalize a CBD working group for biodiversity mainstreaming	CBD 2020d
	Partner with EAT-Lancet Commission for dietary change affecting biodiversity	Willett et al. 2019
Prioritize actions	Strengthen NBSAP reporting	Ulloa et al. 2018
	Expand funding for BIOFIN	www.biodiversityfinance.net
Experiment with pilot projects	Connect national biodiversity goals with local performance incentives	Wang et al. 2020
	Link NBSAP with costs of infrastructure projects	Carter et al. 2020

Transitions are always complex, are subject to uncertainty, and are only amenable to some degree of human guidance (Geels 2019). The key insight in the present article is identifying actions that can accelerate change in the direction one would prefer it to proceed. The Intergovernmental Science-Policy Platform on Biodiversity and Ecosystem Services has initiated a review due in 2022 that will link transformative change research to biodiversity conservation (https://ipbes.net/transformative-change). But conservationists do not have to wait for this report. They can take a step beyond calling for transformative change by reviewing sustainable transitions research and then by experimenting with lessons learned from this work as it may apply to biodiversity conservation. We suggest specific ways to do this below as we focus on the next four steps for injecting change into actions for biodiversity.

## Step 2: Plan for climate change

Climate change is already affecting many aspects of conservation and development. Recent research suggests that by 2030, tropical oceans and forests may be approaching climate tipping points (Trisos et al. 2020), and climate-driven land use will likely affect a majority of global biodiversity hotspots by 2030 (Hannah et al. 2020), even as management response to climate impacts on at-risk species remains inadequate (Delach et al. 2019). There is a fast-growing literature describing climate impacts on biodiversity and how to mitigate them while adapting to present and future conditions (see Arneth et al. 2020, Mahli et al. 2020, Weiskopf et al. 2020).

But the current draft of the Post-2020 Global Framework contains only one target (target 7) that explicitly deals with climate change, and addressing climate is missing from the list of enabling conditions required for implementation of the framework (CBD 2020b). This lack of across-the-board consideration of climate change is problematic because it sets up a scenario where post-2020 goals could be met over the next decade only to fail in the years after 2030 when climate impacts are projected to ramp up (Arneth et al. 2020). To avoid this, climate change should be fully incorporated throughout the Post-2020 Framework. It is beyond the scope of the present article to fully address how this could be accomplished; here, we offer suggestions for several of the Post-2020 Framework targets and provide more details for steps 3–5 below.

Agricultural systems are the primary driver of global biodiversity loss and ecological degradation (Shukla et al. 2019), accounting for about 26% of all greenhouse gas emissions (Poore and Nemecek 2018). Target 9 aims to support the sustainability of biodiversity in agroecosystems, however, in addition to not mentioning climate change, this target only covers the production side of agriculture. In general, this is problematic because researchers have moved beyond focusing only on supply side agricultural intensification (Pretty et al. 2018) and now commonly frame production as but one part of complex food systems (Rockstrom et al. 2020). In particular, this partial framing misses the projection that the largest reductions in agroecosystems emissions out to 2050 are modeled to come from people switching to plant-based diets (a 56% reduction) and diets following improved nutrition guidelines (a 29% reduction; Springmann et al. 2018). Lessons from transitions research suggest that in a world of limited resources, dietary change should be at the top of the list of potential actions to protect biodiversity in food systems. Target 9 should be reframed to include both climate and agroecosystems production and consumption.

Target 14 of the draft Post-2020 Framework focuses on ensuring that negative impacts on biodiversity from production practices and supply chains are reduced by at least 50%. Again, climate change is not addressed despite research showing that the embedded impacts of globalized production have serious carbon emissions consequences (Hull and Liu 2018). A case in point is the greenhouse gas emissions footprint of Brazilian beef exported to the European Union, which almost equals the current carbon mitigation goal of the Bloc (Rajao et al. 2020). Globalization has multifaceted environmental and social footprints and research has identified hotspots of carbon emissions along production and consumption supply chains (Kagawa et al. 2015). These findings could be mapped to biodiversity hotspots to address impacts, but target 14 is silent on this.

The aspirational goal of eliminating unsustainable consumption by assisting people everywhere to understand the value of biodiversity and use this to inform purchasing behavior is the focus of target 15. This is a huge target and needs to be broken down into key sectors; dietary change should be one area of strategic prioritization given its aforementioned role in emissions reductions. Who are the potential institutional partners, and where are the leverage points to support dietary change? One answer is the EAT-Lancet Commission on Food, Planet, Health (https://eatforum.org/eat-lancet-commission). This group has already established a global model diet to encourage transitions toward sustainable, healthy, low emissions eating that the CBD could adopt to help fulfill a critical piece of target 15 (Willett et al. 2019). Given that less than half of all countries have established national dietary guidelines (Herforth et al. 2019), a leverage point for biodiversity conservation and emissions reductions is to work with countries to solidify national dietary standards that approximate the EAT-Lancet standards. Climate cobenefits from these actions could occur through broadening partnerships for biodiversity and building novel national and international cooperative mechanisms for the negotiation of inevitable trade-offs between climate, food systems, biodiversity, public health, and sustainable development goals.

## Step 3: Reframe area-based conservation

Protected areas will always be essential building blocks in efforts to protect biodiversity. But it is clear that, even as additional lands and waters must be conserved to curb accelerating biodiversity loss, traditionally defined protected areas are no longer sufficient by themselves to get this job done (Maxwell et al. 2020). It is time to transform our models of area-based conservation.

To accomplish this, the draft Post-2020 Framework sets a target of at least 30% coverage for lands and waters by 2030 (CBD 2020b; see Dinerstein et al. 2019). This will require elevating biodiversity protection on multiple kinds of lands *outside* legally defined protected areas including key biodiversity areas (Kulberg et al. 2019), other effective area-based conservation management areas (OECMs; lands that may not have biodiversity as a primary goal but are nevertheless managed to include long-term biodiversity outcomes; Donald et al. 2019), contributions from Indigenous peoples’ lands (Garnett et al. 2018), and more.

This expansive goal of 30% or more reveals a key reality of twenty-first century conservation: The dichotomy between protection and use or, more broadly, between nature and people, can no longer serve as an adequate framework for conservation action. This new goal represents a transformative reframing of both conservation and development; there is simply no way area-based conservation can be scaled up to 30% and more of Earth without directly linking management inside protected areas with what happens outside (Dudley et al. 2018). And just as protected areas remain necessary but not sufficient to protect biodiversity, it is also no longer adequate to describe a safe operating space for humanity only in terms of biophysical constraints (Rockström et al. 2009); justice and equity must also factor in to our constructions of safe spaces (Raworth 2017, Fanning et al. 2020). Finally, after decades of work, it is no longer necessary to debate whether conservation is primarily about people or nature. We are all in this together.

The CBD's 2050 Vision for Biodiversity emphasizes people living in harmony with nature, but this creates practical problems on the ground; one conservative estimate shows that at least 170 million people would be living within new conservation areas if biodiversity targets were expanded to 30% of lands (Schleicher et al. 2019). How might these peoples’ livelihood activities support or undermine biodiversity? The same question can be asked of people who may not live near potential new conservation areas but are members of affluent societies with consumption habits that affect biodiversity (Wiedmann et al. 2020).

Along with reframing conservation to acknowledge people and nature, transformative change research shows that accenting coproduction of transdisciplinary knowledge offers an additional way to practice post-2020 conservation (Colloff et al. 2017). Coproduction means supporting researchers from multiple disciplines to combine their talents and work with local actors to create new knowledge to solve biodiversity problems, offering “a powerful tool for stimulating landscape-level systems thinking, integrating diverse knowledge systems, and translating knowledge to implementation” (Nel et al. 2015, p. 183).

We see two areas in which knowledge coproduction can yield near-term benefits for biodiversity. First, conservation scientists agree that lands where biodiversity conservation may not be the primary (legal) goal will play important post-2020 roles. Over the next decade, novel ways to knit traditionally defined protected areas with a host of OECMs, key biodiversity areas, Indigenous people's lands, and other places in which people live and work are going to be required. These diverse, place-specific management arrangements will include multiple objectives, but today in most places they remain abstract and underdeveloped at the scale that is now necessary to create integrated plans. Much more pilot project experimentation is needed to explore the trade-offs inherent between support of local livelihoods and protection of species and ecosystem structures and functions. It is time for the secretariat to expand beyond using groups of experts to review what work has already been done and to create paths for participation for nonstate actors and local people (Tittensor et al. 2019, Lavorel et al. 2020). More diverse groups can work to set up pilot projects in regions around the world that can yield lessons learned about integrating biodiversity protection with human livelihoods on mixed-use lands. Successful pilots could then be scaled up to national and regional levels.

Knowledge coproduction can also advance area-based conservation by integrating climate change into management of protected lands and waters with surrounding areas. The problem is that protected areas as currently configured are static in nature, exhibit poor connectivity, and, as climate change accelerates, redistributions of species and ecosystems will increasingly undermine their biodiversity protection value (Hoffman et al. 2019, Elsen et al. 2020, Lawler et al. 2020). Spatial modeling shows that, as climate shifts, much of high-biodiversity value terrestrial habitat (Mokany et al. 2020), marine habitat (Ramirez et al. 2017), and multiple ecosystem services to support people (Mitchell et al. 2021) will lack protection. And given that impacts will likely create novel ecosystems, notions of ecosystem restoration and recovery will also need to be revised because of new climate conditions (Heger et al. 2019, Prober et al. 2019).

Little of this is covered in the current configuration of the Post-2020 Framework. It is time for the secretariat to engage with these issues and there are several models to consider, including a future-proofing conservation framework (van Kerkhoff et al. 2019), a dynamic conservation approach for marine protected areas (Tittensor et al. 2019), and a climate-informed protected areas network strategy (Stralberg et al. 2020). These innovations can be supported by bringing together biologists, social scientists, and local participants in working groups to discuss and design implementation of new hybrid forms of area-based conservation that account for climate impacts. But what happens if there is weak governance capacity to implement change?

## Step 4: Prioritize biodiversity mainstreaming

Mainstreaming means incorporating biodiversity goals into all levels of local and national environmental planning so that they become a standard feature of decision making (Whitehorn et al. 2019). The secretariat has long recognized that the CBD's objectives would be “impossible to meet” until this goal is achieved (CBD 2005), and there have been multiple attempts to strengthen mainstreaming. But it continues to be out of reach for most parties. A major reason for this is that biodiversity conservation has not typically addressed the social values and cultural, and economic roots that impede mainstreaming (Wyborn et al. 2020). Another reason is that mainstreaming is more about politics and less about biology.

Most countries have basic environmental rule of law on paper, but application, compliance, and enforcement are often lacking (UNEP 2019). Mainstreaming is poorly practiced in many governments and remains rare in private sector planning. There are dramatic differences between countries in institutional and resource capacity to support mainstreaming. And CBD implementation processes are voluntary, so parties often act to maintain control where mainstreaming might reduce space for sovereign decision making (Dubash 2020).

In fact, the CBD and others have identified multiple solutions to mainstreaming conundrums. Since 2012, eight reviews of mainstreaming have been conducted by various international bodies, and they all restate many of the same problems and steps to fix them (Chandra and Idrisova 2011, CBD 2020c). Common solutions include providing disaggregated data on biodiversity to the many countries that lack them, establishing national indicators and monitoring capacity for parties to track changes in biodiversity, supporting biodiversity communication networks, increasing engagement with nonstate actors (especially businesses), promoting more citizen participation, and reducing negative incentives and boost funding (Whitehorn et al. 2019, CBD 2020d, 2020e, Han et al. 2020).

With broad agreement on problems and solutions, the question becomes what near-term mainstreaming strategies might begin to reverse decades of limited progress? One obvious place to look for improvement would be the CBD's primary mainstreaming tool, the National Biodiversity Strategies and Action Plans (NBSAPs). As of 2020, the great majority of parties had submitted NBSAPs to the secretariat, but only 44% had adopted them as policy, and just 39% of countries had actually integrated biodiversity into national planning (CBD 2020f). Sustainable transitions research shows that it is best to focus on national mainstreaming projects while encouraging countries to strengthen links to local-level efforts to implement changes (Heikkila and Gerlak 2019, Huge et al. 2020). For example, China is experimenting with performance incentives for local officials if they meet national biodiversity targets (Wang et al. 2020). But the secretariat has not provided a robust framework to parties to work with, nor has it strategically prioritized where limited mainstreaming resources should go.

Prioritizing mainstreaming support would be politically contentious if the CBD was perceived as picking winners and losers, but we see four opportunities to act that can build common ground. First, because the CBD's attempts to establish an online clearing house of biodiversity information for parties have been unsuccessful, the secretariat should continue to strengthen support for other efforts including the NatureServe network in the United States and Canada (www.natureserve.org/natureserve-network), the Integrated Biodiversity Assessment Tool (www.ibat-alliance.org/), and the new Biodiversity Health Index (Soto-Navarro et al. 2020). Second, a formal mainstreaming working group composed of parties, experts, business actors, and nonstate participants should replace the CBD's informal group to serve as a general incubator for innovative ideas. This group's first action should be aimed at strengthening the CBD's NBSAP reporting template so that gaps between plans and convention goals are reduced. Discussion to do this is underway, however, it remains unclear if and when this process will be completed. For parties that have not yet submitted NBSAPs, the secretariat should systematically investigate barriers to delivery, and be less hesitant to employ peer accountability (Ulloa et al. 2018).

Third, facing increasing climate impacts along with the greatest global growth in infrastructure in history (zu Ermgassen et al. 2019), it is imperative that the CBD encourage parties to focus on projects that present near-term threats to biodiversity. This work could yield project-specific action plans that would factor in costs of environmental, social, and climate impacts over the lifetime of proposed developments. Countries for potential focus include Democratic Republic of Congo, New Guinea, Indonesia, Ecuador, Malaysia, Mexico, Peru (Laurance et al. 2014, Johnson et al. 2020), and multiple nations engaged with China's Belt and Road Initiative (Hughes 2019, Carter et al. 2020). Then the secretariat could sponsor pilot projects with those countries that are ready to mitigate (or reduce) infrastructure projects, linking this work to countries’ extant NBSAPs.

Finally, there already exists a working example of mainstreaming: The United Nations Development Programme's Biodiversity Finance Initiative (BIOFIN, www.biodiversityfinance.net). BIOFIN engages government ministries and financial actors to create/implement Biodiversity Financial Plans linked to existing NBSAPs. This successful program has been implemented in 36 countries (of which 11 are megadiverse), and funding should be scaled up to facilitate general biodiversity mainstreaming.

These actions may not appear to be particularly transformative, but they address all three of the necessary preconditions for successful mainstreaming: increasing knowledge accessibility, resources, and functional institutions (Milner-Gulland et al. 2021). Collectively, over time, they can move parties and the secretariat toward new behaviors that can become business as usual for sustainable transitions. The truth about mainstreaming is that the CBD cannot transform social values or sovereign government actions toward protecting biodiversity over a short-term time scale. Nor have there been available the financial resources to do so.

## Step 5: Scale up resource mobilization

Chronic underfunding has plagued the CBD since its inception, so increasing resources for biodiversity conservation is critical for transformative change. The numbers are stark. Funding for biodiversity (average over 2015–2017) is estimated to be US$78–$91 billion per year (OECD 2020). The CBD-estimated costs of implementing the Aichi targets were US$150–$440 billion per year (CBD 2020g). Looking ahead, costs to expand terrestrial area-based conservation by 30% range around US$100 billion per year (certainly a minimal estimate; Dinerstein et al. 2019). Marine protected areas may require an additional US$174.2 billion per year to 2030 (Johansen and Vestvik 2020). We also know that lands outside formally protected areas will play pivotal roles in post-2020 conservation, but there are no cost estimates specific to embedding biodiversity protection within management of these areas.

To trigger change, the CBD's vision of resource mobilization should be reframed away from seeking ever more resources to close ever wider funding gaps and toward economic transformation that greatly reduces need for biodiversity funding. Global estimates for perverse environmental subsidies from governments alone hover around US$500 billion per year (OECD 2020), and although no one expects these subsidies to be reduced overnight or be used wholesale to fund conservation, it is widely recognized that they can play a role in minimizing the need for (new) resources to manage ongoing loss of biodiversity (Dempsey et al. 2020).

New funding from business, development banks, and governments is also necessary to make manifest a reframed vision for resource mobilization. The World Economic Forum recently reported that more than 50% of global GDP is moderately to highly dependent on biodiversity, and awareness of implications of biodiversity loss for business may be reaching some critical mass (WEF 2020). But the CBD has had no fully operational resource mobilization plan since 2015 (CBD 2020g). The secretariat has gathered recommendations from an expert group on resource mobilization for discussion at COP 15 (CBD 2020h), and research exists that uses a country's developmental status to yield a socioecological design for biodiversity funding under the CBD (Droste et al. 2019). Stronger links to climate change may also strengthen support for biodiversity funding (Gardener et al. 2020). These efforts should spur strategies to increase collaboration with and funding from multidevelopment banks, private businesses, and donors, despite financial fallout from the SARS-CoV-2 virus creating new challenges.

Funding aside, increasing post-2020 resources for biodiversity is fraught with practical and political challenges. Sustainable transitions research shows that when resources remain scarce, projects should be prioritized so that limited funds may be spent efficiently. But in efforts from 2010–2020 to meet Aichi target 11, management effectiveness and money to pay for it lost ground to the goal of increasing the quantity of protected areas (Gill et al. 2017, Coad et al. 2019). This must change in the post-2020 era; the CBD's aspirational goals to protect 30% of Earth's lands and waters should not result in underfunded and ineffectively managed mixed-use conservation areas. Instead, if conservation for people and nature is to successfully expand despite limited resources, funders and practitioners must ask *How great are the threats to biodiversity here?* , *How much will it cost to effect change?* , *How long will action take?* , and *How best can we work with local people as equals in design and decision making?* These practical questions can serve as the basis to solve problems, prioritize investments, and sort through inevitable tradeoffs between resource mobilization benefits and costs (Kuempel et al. 2020, Yang et al. 2020).

The politics of scaling up resources for transformative change through greater engagement with private sector and nonstate actors has its own set of costs and benefits. The secretariat will be called on to manage tensions between investors demanding level playing field monitoring and national compliance standards for biodiversity projects. There are also parties and nonstate actors who will be slow to eliminate negative incentives and local stakeholders who will need additional financial capacity to be able to take action. These challenges apply to many resource mobilization issues: payments for ecosystem services, biodiversity offsets, markets for green products, and climate change mitigation (CBD 2020h). Given that transformative change is often stymied by top-down management, resource mobilization may be better implemented using multiple working groups to identify nations that are willing partners, specific institutions within countries that are best positioned to act, and projects that garner grassroots support (Abson et al. 2017). It is not yet clear how much political will and institutional capacity international actors such as the CBD have to work with a greater diversity of nonstate groups and local people (Griffiths et al. 2020). It is clear that the secretariat cannot accomplish post-2020 resource mobilization work without bold new actions.

## Change agents for biodiversity

The five steps outlined above will transform the very nature of work under the CBD. A convention based on voluntary implementation by nation states, facilitated by international administrators aided by advice from a relatively narrow range of scientists and experts, must now open up to a broader range of participants from multidevelopment banks and businesses (Smith et al. 2020) to Indigenous peoples (Ban et al. 2018).

To accommodate these transitions, biodiversity advocates must examine what their potential roles may be in supporting transformative change. For the secretariat, transformative change means becoming a more effective and efficient agent for on-the-ground implementation of biodiversity conservation. This means doing much more to connect provision of biodiversity data with prioritized pilot projects that include timelines/milestones for progress, and dedicated funding. The convention may serve as an aspirational role model to reframe conservation for nature and people. However, despite much discussion stretching back for years, the secretariat has yet to coproduce plans with UN colleagues to directly link the CBD's goals with the SDGs (Rogalla von Bieberstein et al. 2018, CBD 2019), although this is under discussion in preparation for COP 15 (CBD 2020h). The secretariat also must do more work to link the CBD with the Intergovernmental Panel on Climate Change.

For researchers, transitions require that more projects are coproduced with transdisciplinary partners so that science, social issues, and the politics of change are all addressed. Change is already occurring as more biologists work with transdisciplinary teams and join biophysical with social and political perspectives; recent examples include examining cultural equity as well as biological flows in ecosystem services (Kleemann et al. 2020), exploring the inclusion of biodiversity-driven economic scenarios into CBD planning (Otero et al. 2020), evaluating ocean protection through a social justice lens (Cisneros-Montemayor et al. 2020), and reviewing social equity across conservation research (Friedman et al. 2018). Still, most biodiversity research remains focused on describing biophysical threats to nature with relatively little work being done on design and implementation of conservation actions (Williams et al. 2020). The gains in our baseline understanding of biodiversity need to be matched by the willingness to design and publish transdisciplinary solutions to protect it.

For the parties, the biodiversity crisis (along with lagging efforts to meet climate change and SDG goals) demands that governments stop trading away conservation actions now for aspirational ambitions in the future. We are not sanguine about expectations for short-term change in nation-state behavior toward biodiversity, despite the ideals of diverse participation, justice, gender equity, political transparency, and more that are embedded in the CBD and the draft Post-2020 Framework. In the time of the SARS-CoV-2 virus, learning how to stave off global economic meltdown comes first, and rebooting progress toward the SDGs and climate action will likely take precedence over biodiversity conservation. The virus provides all the more reason to foreground messages that link human well-being to biodiversity conservation and human development to a healthy, diverse planet through the SDGs.

In the present article, we must emphasize the uncertainty inherent in transformative change. The future is not preordained; 170 of 196 countries have delivered NBSAPs under the CBD, and nudging nation states toward more sustainable behavior will be accomplished through many steps entailing much experimentation. Like climate issues, biodiversity conservation for people and nature is a driver of transformative change in social values and behavior, but what looks like a crisis to conservationists has not yet inspired behavioral change in people or a critical mass of decision makers to act much beyond signing on to the aspirational aims of the CBD.

## Conclusions

History shows that transformations often occur after initial disturbances create “windows of opportunity” for new values, behaviors, and institutions to emerge (Otto et al. 2020). By many measures, including the presence of the SARS-CoV-2 virus, windows are opening now. Transitions research reveals that these openings invite strategies to help quicken the pace of change: innovative reframing, knowledge coproduction, coalition-led pilot projects, more diverse and equitable participation in conservation, and more. Emergent change, however, is incremental; consider the decades-long evolution of the CBD. Some progress has been made toward achieving the CBD's strategic goals, but the loss of biodiversity has proceeded much more rapidly. The convention by itself was never designed to shoulder the burden of societal transitions toward sustainability. But it can be revitalized to better support and (even) accelerate change if the secretariat, parties, conservation scientists, and advocates are willing to use new knowledge to foster the transformations that people and nature now depend on.

## Author Biographical

R. Edward Grumbine (ed.grumbine@gmail.com) is a biodiversity policy analyst who recently held a fellowship through the Chinese Academy of Sciences President's International Fellowship Initiative at the Centre for Mountain Futures, Kunming Institute of Botany, in Kumming, China. Jianchu Xu (jxu@mail.kib.ac.cn) is the director of the East and Central Asia Office at the World Agroforestry Centre, in Kunming, China, and is the director of the Centre for Mountain Futures and a professor at the Kunming Institute of Botany, Chinese Academy of Sciences, in Kunming, China.
